# Isolated trochlear nerve (cranial nerve IV) palsy in a patient with carotid-cavernous fistula 

**DOI:** 10.1259/bjrcr.20180028

**Published:** 2018-09-08

**Authors:** Aditya Durgam, Huda Hussain, Austin S. Nakatsuka, Karthikram Raghuram

**Affiliations:** 1 Department of Radiology, University of Texas Medical Branch , Galveston, TX

## Abstract

Isolated fourth cranial (trochlear) nerve palsy is an uncommon occulomotor nerve palsy typically secondary to ischemia, inflammation/infection, or mass effect. Due to its positioning within the cavernous sinus, pathology of the deep cerebral venous system would theoretically predispose to palsy of this nerve. We present a case of a patient presenting with isolated trochlear nerve palsy in the setting of angiographically-confirmed direct carotid-cavernous fistula.

## Report of case

**Figure 1.**
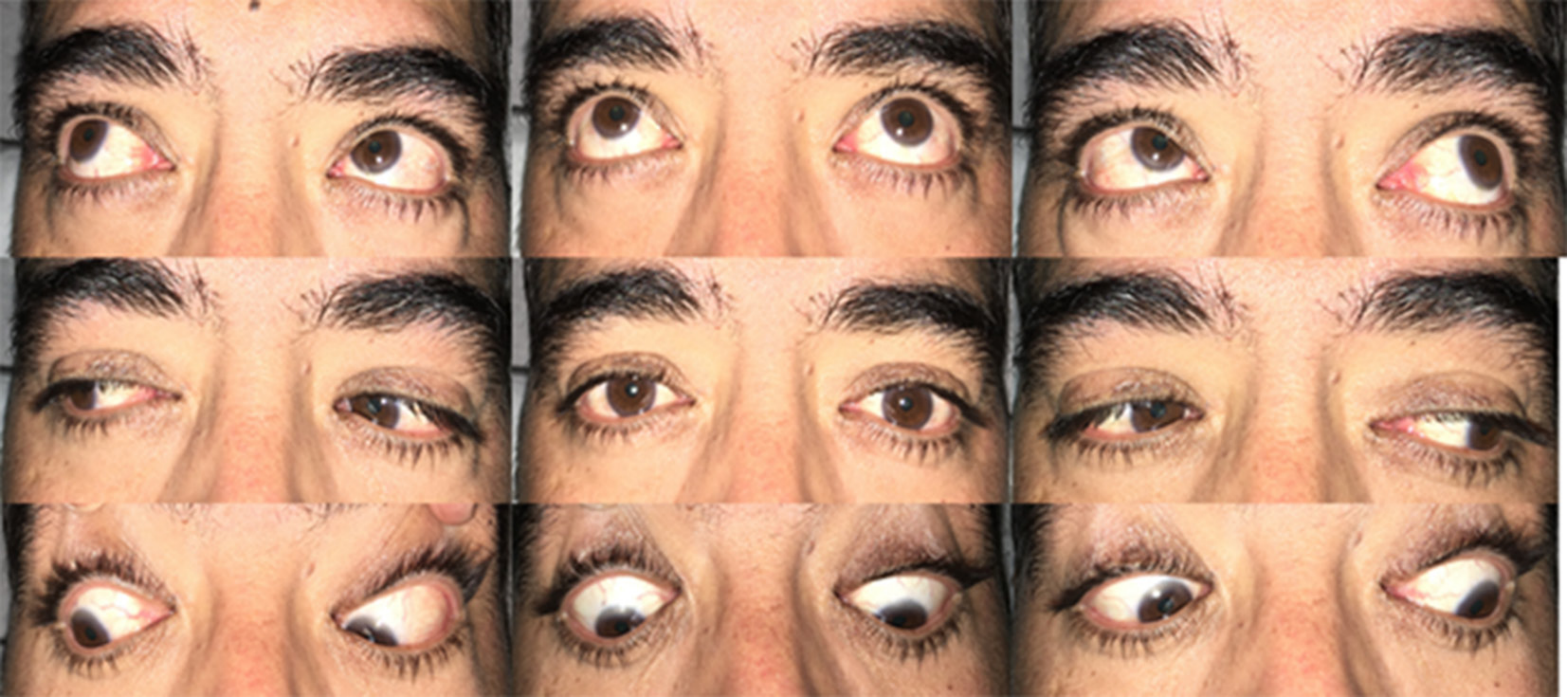
Pre-operative positions of gaze.

A 51-year-old male presented to the neurology clinic with 1 week history of right-side retrobulbar pain. Initial clinical examination revealed no significant findings. The patient continued to have right retrobulbar pain not amenable to over the counter pain medication. 1 week later the patient woke up with binocular vertical diplopia, constant and worsened on downgaze (Figure 1). He reported no changes in visual acuity, irritation, flashes or floaters. No history of trauma was identified. The past medical history is negative for hypertension or diabetes and reveals no risk factors for vasculopathy. The ophthalmological examination was positive for mild depression and intorsion of right eye on downward gaze consistent with fourth nerve palsy.

Neurological examination and assessment of the remaining cranial nerves were not significant. Ophthalmology recommended testing for thyroid eye disease as well as evaluation for acetylcholine-receptor autoantibodies to rule out Myasthenia Gravis, both of which were negative.

**Figure 2.**
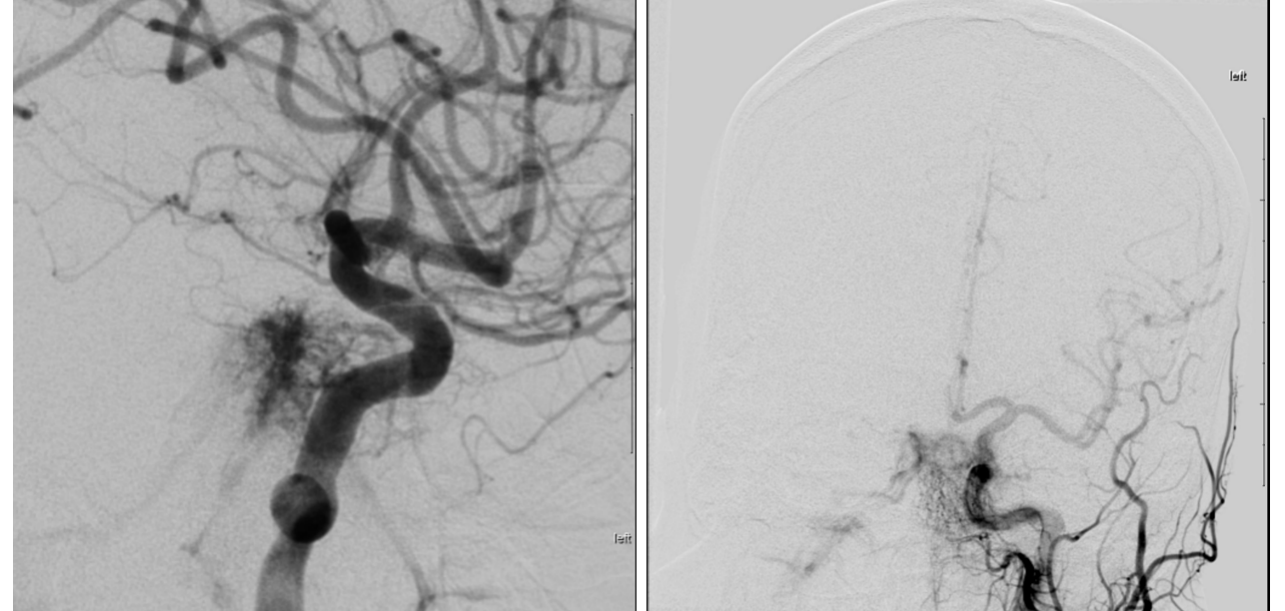
Pre-operative cerebral angiography with left internal carotid artery selective injection in magnified oblique (left) and Anterior-Posterior (right) views, showing early filling of the cavernous sinus consistent with carotid-cavernous fistula.

Subsequently, MR imaging of the brain and orbit was ordered and proved inconclusive. CT angiography of the head was ordered and demonstrated an incidental finding of an enlarged vein in the anterior interhemispheric fissure draining into a prominent inferior sagittal sinus. As no specific vascular abnormality was suggested on CT angiography, no further cross-sectional imaging was performed. On the basis of the clinical presentation and physical examination, cerebral angiography was then performed and revealed bilateral indirect carotid-cavernous fistulae with the predominant feeders arising from the bilateral ascending pharyngeal arteries and small feeders from bilateral cavernous internal carotid arteries. (Figure 2).

The findings and treatment options were discussed with the patient who preferred to proceed with embolization. Patient was discharged and scheduled for embolization 2 weeks later.

**Figure 3.**
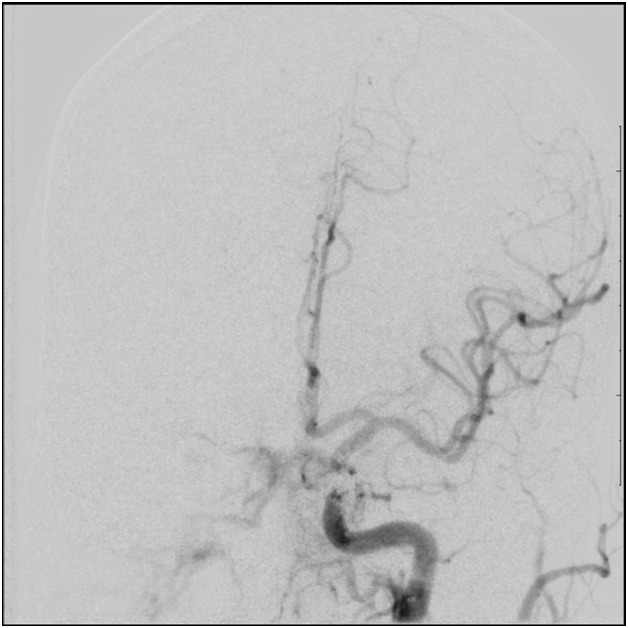
Post-operative cerebral angiography with left internal carotid artery selective injection in the Anterior-Posterior view showing a cavernous sinus filling defect at the level of the embolized fistula.

Right transfemoral arterial access was obtained with a 4 french catheter system to access the right external carotid artery, while left transfemoral venous access was achieved for 6 french Neuron 070 guide catheter placement in the right internal jugular vein. After initial attempts to catheterize the right inferior petrosal sinus proved ineffective, the left internal jugular vein was catheterized and a Mirage wire and Apollo microcatheter were used to access the left inferior petrosal sinus. A microcatheter angiogram was performed to confirm positioning within the left cavernous sinus. Onyx was used to embolize the cavernous sinus while systemic heparinization was performed to maintain Activated Clotting Time>250. At the completion of the procedure, only minimal residual shunting to the right cavernous sinus was observed (Figure 3).

1 day post-procedure assessment revealed resolution of the fourth cranial nerve palsy but unfortunately the patient developed right sixth nerve palsy which was thought to be secondary to nerve edema from pressure effect of the embolic material. The patient was prescribed oral prednisone and vitamin B6. Ophthalmological evaluation 6 months later revealed progressive improvement of the left eye esotropia. A follow-up angiogram showed complete resolution of the fistula with no residual shunting.

## Discussion

The six extraocular muscles are innervated by three separate cranial nerves: the oculomotor (third cranial) nerve is responsible for the function of the superior, inferior, and medial rectus, inferior oblique, and levator palpebrae superioris muscles; the trochlear (fourth) nerve supplies the superior oblique; and the abducens (sixth) nerve supplies the lateral rectus muscle. All three of these nerves course through the cavernous sinus on their path to the orbit, providing an inconvenient vulnerability for the transmission of cerebral or systemic infection, inflammation, and vascular pressure differentials capable of perturbing the extraocular neuromuscular system.

Isolated trochlear nerve palsy has been reported in the literature, with the most common identified etiologies being microvascular ischemia and other causes including trauma, vascular disease, and tumors (such as schwannomas).^1^ Other identified causes have included herpes zoster ophthalmicus, tuberculous meningitis, neuromyelitis optica, Lyme neuroborreliosis, Tolosa-Hunt syndrome, and subarachnoid hemorrhage.^2–6^ Aneurysm of the cavernous segment of the internal carotid artery have also been reported as a cause of isolated cranial nerve palsy, with one case report suggesting visit-to-visit variability in systolic blood pressure as the etiology of intermittent palsy, which resolved with blood pressure control.^7^ Numerous authors, such as Kung and Murchison, suggest MR imaging to be routinely performed in individuals > age 50 who present with unilateral cranial nerve palsy to look for causes other than microvascular ischemia.^8,9^

To our knowledge, our report represents the first reported case of isolated cranial nerve palsy secondary to a carotid-cavernous fistula. By definition, Carotid Cavernous Fistula is an abnormal connection of the internal, external carotid arteries or their branches, and the cavernous sinus. Based on Barrow classification, there are four types based on the communicating artery: Type A, direct communication with ICA; Type B, with the meningeal branches of ICA; Type C, meningeal branches from external carotid artery; Type D, meningeal branches from ICA and external carotid artery.^10^

MR imaging performed to evaluate for the common causes (including ischemia and mass) revealed extraorbital findings of vascular engorgement which are seen more often with carotid cavernous fistula formation, which were confirmed with conventional four-vessel digital subtraction angiography.

In regard to the procedural technique implemented in our case, the decision to use onyx embolization material rather than coil embolization was multifold. As the lesion was diffuse, use of coils would likely have required a larger volume of coils to occlude the fistula with subsequent increased mass effect upon the cranial nerves as they traverse the cavernous sinus. Onyx embolization was used primarily for this reason as well as to achieve penetration to the contralateral (right) cavernous sinus with the least mass effect. Additionally, no contralateral common carotid artery compression nor double balloon catheter techniques were employed during the procedure.

In our patients’ case, although the right fourth nerve palsy resolved after embolization, sixth nerve palsy subsequently developed. In none of the reported cases of isolated fourth nerve palsy was a similar subsequent cranial nerve palsy reported after treatment, although only one was due to a cerebrovascular anomaly (arteriovenous fistula right tentoral incisura, which was embolized with resolution of diplopia).^11^

A further review of the literature suggests that paradoxical worsening of the incident cranial nerve palsy has been reported in as many as 12.5% of patients undergoing treatment for cavernous dural arteriovenous fistula.^12–15^ Postulated causes of this paradoxical worsening or new cranial nerve palsies may be due to progressive thrombosis of the superior ophthalmic vein, the cavernous sinus, or their tributaries; clot propagation; mass effect due to the use of coils; or direct injury to the nerve by coils or injury due to microwire/microcatheter manipulation. Additionally, the sixth cranial nerve could be injured in Dorello’s canal as the inferior petrosal sinus is catheterized.^13,16–18^

Given relevant anatomy and, development of other cranial nerve abnormalities should be considered and discussed with the patient prior to treatment of the causative carotid-cavernous fistula. After development of the post-procedural sixth nerve palsy, the patient was discharged with oral prednisone. On ophthalmological follow-up 6 months later, the patient’s esotropia had improved although continued follow-up is ongoing.

## Learning points

- Neural structures within the cavernous sinus, including the occulomotor, abducens, ophthalmic and maxillary divisions of the trigeminal, and trochlear nerves, are susceptible to injury in the setting of cavernous sinus pathology.
- Carotid-cavernous fistula should be considered in the differential diagnosis for isolated unilateral cranial nerve palsy.
- Treatment of carotid-cavernous fistulae may in turn result in iatrogenic cranial nerve palsy, which, in our case, resolved over time.
